# Profiles of instrumental, emotional, and informational support in Chinese breast cancer patients undergoing chemotherapy: a latent class analysis

**DOI:** 10.1186/s12905-021-01307-3

**Published:** 2021-04-29

**Authors:** Tingting Cai, Qingmei Huang, Changrong Yuan

**Affiliations:** School of Nursing, Fudan University, 305 Fenglin Road, Shanghai, 200032 China

**Keywords:** Instrumental support, Emotional support, Informational support, Chemotherapy, Breast cancer

## Abstract

**Background:**

To date, few studies have assessed social relationships in patients with breast cancer during their chemotherapy process. This study aimed to explore profiles of instrumental, emotional, and informational support in Chinese breast cancer patients undergoing chemotherapy.

**Methods:**

In a cross-sectional study performed between September 2018 and September 2020 in China, 638 patients with breast cancer completed demographic information questionnaires, the PROMIS-Social Relationships Short Forms, the PROMIS-Anxiety Short Form, and the PROMIS-Depression Short Form. Analysis of variance and chi-square tests were performed to examine between-group differences in demographic characteristics, anxiety, and depression outcomes across the identified latent classes. Multinomial logistic regression was performed to identify the correlation of significant variables among the identified classes.

**Results:**

Three profiles of social relationships were identified: Class 1-low social relationships group (14.3%), Class 2-high instrumental support, medium emotional and informational support group (24.6%), and Class 3-high social relationships group (61.1%). The findings revealed the heterogeneity of instrumental, emotional, and informational support in Chinese breast cancer patients undergoing chemotherapy, which was significantly correlated with educational background, monthly family income, health insurance, and employment status. Additionally, patients with low instrumental, emotional, and informational support were more likely to report high levels of anxiety and depression.

**Conclusions:**

When conducting interventions to promote social relationships during the chemotherapy process, healthcare providers should consider the sociodemographic characteristics, anxiety levels, and depression symptoms of patients with breast cancer and identify high-risk patients for tailored interventions.

## Background

Globally, breast cancer is the most commonly diagnosed cancer in women [1]. Early diagnosis with advanced therapeutic strategies has led to a steady rise in long-term survivors. Despite significant improvement in survival, patients with breast cancer are faced with multiple challenges, including psychological and physical symptoms and changes in interpersonal relationships [2, 3]. Cancer-related issues and lifestyle changes may cause altered relationships throughout diagnosis, treatment, and follow-up but have not been stressed in this particular context [4–6]. Frequent outpatient visits and medical treatments significantly reshape a woman’s social relationships after being diagnosed with breast cancer [7, 8]. Therefore, these patients have unique social relationships needs to receive support from their family, friends, peers, or healthcare providers. Moreover, having strong and stable social relationships is essential to promote the health outcomes of patients with breast cancer [9]. Kroenke et al. [10] discovered that socially isolated individuals are twice as likely of dying from breast cancer as socially integrated women. Crookes et al. [11] reported that women with few social networks were more likely experience a breast cancer relapse than patients with large social networks. As receiving chemotherapy is a common and stressful event in this population that could cause anxiety and depression, it is therefore vital to assess the social relationship level of patients undergoing chemotherapy and identify predictors for high-risk groups.

Instrumental, emotional, and informational support are important forms of social relationships [12, 13]. Instrumental support is provided by someone who helps with one’s activities of daily life, emotional support involves perceiving being cared for and valued, and informational support involves individuals providing valuable advice [12, 13]. To date, few studies have assessed instrumental, emotional, and informational support in patients with breast cancer during their chemotherapy process. These patients might have varied social needs due to differences in their demographic and clinical characteristics. Therefore, it is vital to assess and address these needs from the patient’s self-reported perspective.

In recent years, the patient-reported outcomes measurement information system (PROMIS) has been widely launched. The PROMIS is a set of self-reported tools based on item-response theory that allows researchers to ascertain a person’s symptoms or functioning using minimum responses without losing precision and maintaining comparability across a wide array of disease groups [14]. The PROMIS comprises physical, mental, and social health domains and is the preferred method for assessing subjective perceptions, including in the social relationships domain [14]. Accumulating evidence has suggested that social relationships are important determinants of health outcomes [13, 14]. Given the importance of social relationships, obtaining a patient’s self-reported perspective is necessary for understanding their social needs. Studies have rarely advanced person-centered approaches in this field. Latent class analysis (LCA) is a commonly used person-centered method to identify individuals who have similar features and classifies similar individuals into corresponding latent classes. Therefore, LCA is an ideal approach for exploring social relationship profiles [15]. Considering the potential influence social relationships have in this population, this study utilized the PROMIS-Social Relationships Short Forms as the primary measurement, aiming to identify profiles of self-reported instrumental, emotional, and informational support through LCA in Chinese breast cancer patients undergoing chemotherapy and to explore the differences in sociodemographic characteristics in addition to anxiety and depression outcomes.

## Methods

### Study design and samples

This cross-sectional study was based on the STROBE checklist. It utilized nonprobability, consecutive convenience series sampling to assimilate patients from tertiary hospitals in Shanghai (mainland China) between September 2018 and September 2020. The following inclusion criteria for the patients were applied: (1) aged ≥ 18 years; (2) diagnosed with breast cancer; (3) undergoing chemotherapy; and (4) able to read and understand Chinese. Patients with severe psychiatric disorders that prevented effective communication were excluded. It was easier to contact the patients during their clinical visit, so all the patients were recruited during hospitalization.

### Research instruments

A set of questionnaires consisting of demographic and clinical characteristics and several valid PROMIS measures available in Chinese were utilized. The licenses of the PROMIS-Social Relationships Short Forms, the PROMIS-Anxiety Short Form, and the PROMIS-Depression Short Form were obtained from the PROMIS National Center-China in this study. Completion of the questionnaires required 20–30 min.

#### Demographic and clinical characteristics

The demographic information questionnaire collected information on age, religion, marital status, childbearing history, educational background, menstrual status, living style, employment status, monthly family income, health insurance and cycles of chemotherapy.

#### PROMIS-Social Relationships Short Forms

The PROMIS-Social Relationships Short Forms contain 4-, 6-, or 8-item questionnaires on instrumental support, emotional support, and informational support [16]. The Chinese version of the 4-item PROMIS-Emotional Support, Informational Support, and Instrumental Support Short Forms were utilized in this study. For each short form, items are scored using a 5-point Likert-type scale within a 7-day recall period: 1 = never, 2 = rarely, 3 = sometimes, 4 = often, 5 = always [16]. The total scores of each short form range from 4 to 20, with higher scores showing higher social relationships in the corresponding subdomain. Based on the 2000 US census, the PROMIS T score metric was calibrated using the general US population as a reference, yielding a mean of 50 and a standard deviation (SD) of 10 on a T scale [16]. The Chinese version of the PROMIS-Social Relationships Short Forms has been validated in patients with breast cancer and demonstrated satisfactory reliability and validity [17]. The Cronbach’s alphas of the PROMIS-Emotional Support, the PROMIS-Informational Support, and the PROMIS-Instrumental Support Short Form in this study were 0.92, 0.93, and 0.94, respectively.

#### PROMIS-Anxiety Short Form

The Chinese version of the 8-item PROMIS-Anxiety Short Form was used to assess anxiety symptoms [17], and the responses refer to a 7-day recall period and are rated on a 5-point Likert-type scale ranging from 1 (never) to 5 (always). The scores are summed with a minimum score of 8 (no anxiety) and a maximum score of 40 (severe anxiety) [18]. The raw scores were then converted to standardized T scores (mean = 50, SD = 10). Higher scores reflect a greater level of anxiety. Cronbach’s α was 0.90 for the scale within the sample.

#### PROMIS-Depression Short Form

Depression was measured using the Chinese version of the 8-item PROMIS-Depression Short Form [17]. Respondents are asked to rate how frequently they experienced depression in the past 7 days, and their responses are rated on a 5-point Likert response scale (never, rarely, sometimes, often, and always) [19]. Total scores range from 8 to 40, with higher scores indicating severe depression [20]. The raw scores are also transformed into standard T scores (mean = 50, SD = 10). Cronbach’s α was 0.88 for this scale within the sample.

### Ethical considerations

The ethics committee of the Institutional Review Boards of Fudan University Cancer Hospital (no 1810192-22) and Fudan University Zhongshan Hospital (no 2020-076R) reviewed and approved this study. Data were collected by trained nurse professionals. First, the aim and process of the study were explained orally before the investigation. Trained nurse professionals also explained the voluntary nature of the investigation, as well as the anonymity and confidentiality of the data, to each participant. Participants were informed that they could withdraw from the study at any stage without negative consequences. Informed consent was signed by each participant, and the questionnaires were completed either on paper or using a web-based system. Questionnaires were retrieved immediately, and only the researchers had access to the data.

### Statistical analysis

Data were analyzed using SPSS version 21.0 (IBM Co., 2016) and Mplus version 7.4 (Muthen and Muthen, 1998–2017). Initially, descriptive statistics were performed for all the measured variables using frequencies, percentages, means, and SDs. To identify the optimal latent class solution, a series of LCA models with an increasing number of latent classes were constructed using Mplus (1–6 classes). Lower Akaike Information Criterion (AIC), Bayesian Information Criterion (BIC) and adjusted Bayesian Information Criterion (aBIC) values, higher entropy values, and no classes with too few members (e.g., less than 5% of the sample) indicates a better fitting model [21]. In addition, the Lo–Mendell–Rubin (LMR) test *P* value and the Bootstrap Likelihood Ratio Test (BLRT) *P* value were compared between the solution with k classes and the solution with k − 1 classes, and the statistically significant values suggested that the wider solution (k classes) fit the data better [21]. Therefore, the number of classes was selected based on lower information criterion scores (AIC, BIC, and aBIC), higher entropy score, and significant LMR and BLRT *P* values [21]. Additionally, meaningful clinical interpretability was considered.

Subsequently, analysis of variance and chi-square tests were performed to examine between-group differences after identifying the latent social relationships classes. Analysis of variance was used to assess the differences between the continuous variables such as age, anxiety and depression outcomes. Additionally, chi-square tests were used to identify the categorical variables that distinguished the classes. Based on the LCA results, logistic regression was performed to explore the influence of demographic variables on the profiles of social relationships. All statistical tests were two-tailed, and a *P* value of < 0.05 was considered statistically significant.

## Results

### Characteristics of the sample

A total of 705 patients with breast cancer were approached, and 650 were enrolled in the study (response rate, 92.20%). A total of 638 patients completed all the questionnaires. The major reason for refusing was being overwhelmed with cancer treatment. The average age of the respondents was 48.6 ± 10.1 (range = 23–77) years. Most of the respondents were without religion (91.5%), were married (93.9%), had a childbearing history (97.2%), were premenopausal (52.7%), had completed high school education (33.0%), lived with family (94.2%), were employed (45.8%), had a monthly family income < ¥ 6000 (50.2%), had employee health insurance (40.8%), and received 4–6 cycles of chemotherapy (42.3%) (Table 1).

**Table 1:** Sample characteristics of the participants

Variables	Frequency	Percent
Age		
20–29	16	2.5
30–39	106	16.6
40–49	222	34.8
50–59	207	32.6
≥ 60	87	13.5
Religion		
Yes	54	8.5
No	584	91.5
Marital status		
Single	13	2.0
Married	599	93.9
Divorced	15	2.4
Widowed	11	1.7
Childbearing history		
Yes	620	97.2
No	18	2.8
Menstrual status		
Premenopausal	336	52.7
Postmenopausal	302	47.3
Education background		
Primary school or below	162	25.4
Secondary school	119	18.7
High school	211	33.0
University or above	146	22.9
Lifestyle		
Living alone	18	2.8
Living with family	601	94.2
Living with others	19	3.0
Current employment		
Employed	292	45.8
Retired	160	25.1
Unemployed	186	29.1
Monthly family income (¥)		
≤ 6000	320	50.2
> 6000	318	49.8
Medical insurance		
Free medical insurance	4	0.6
Employee health insurance	260	40.8
Rural health insurance	343	53.8
Without health insurance	31	4.8
Cycles of chemotherapy		
1–3 cycles	247	38.7
4–6 cycles	270	42.3
> 6 cycles	121	19.0

### Latent classes of social relationships

**Fig. 1:**
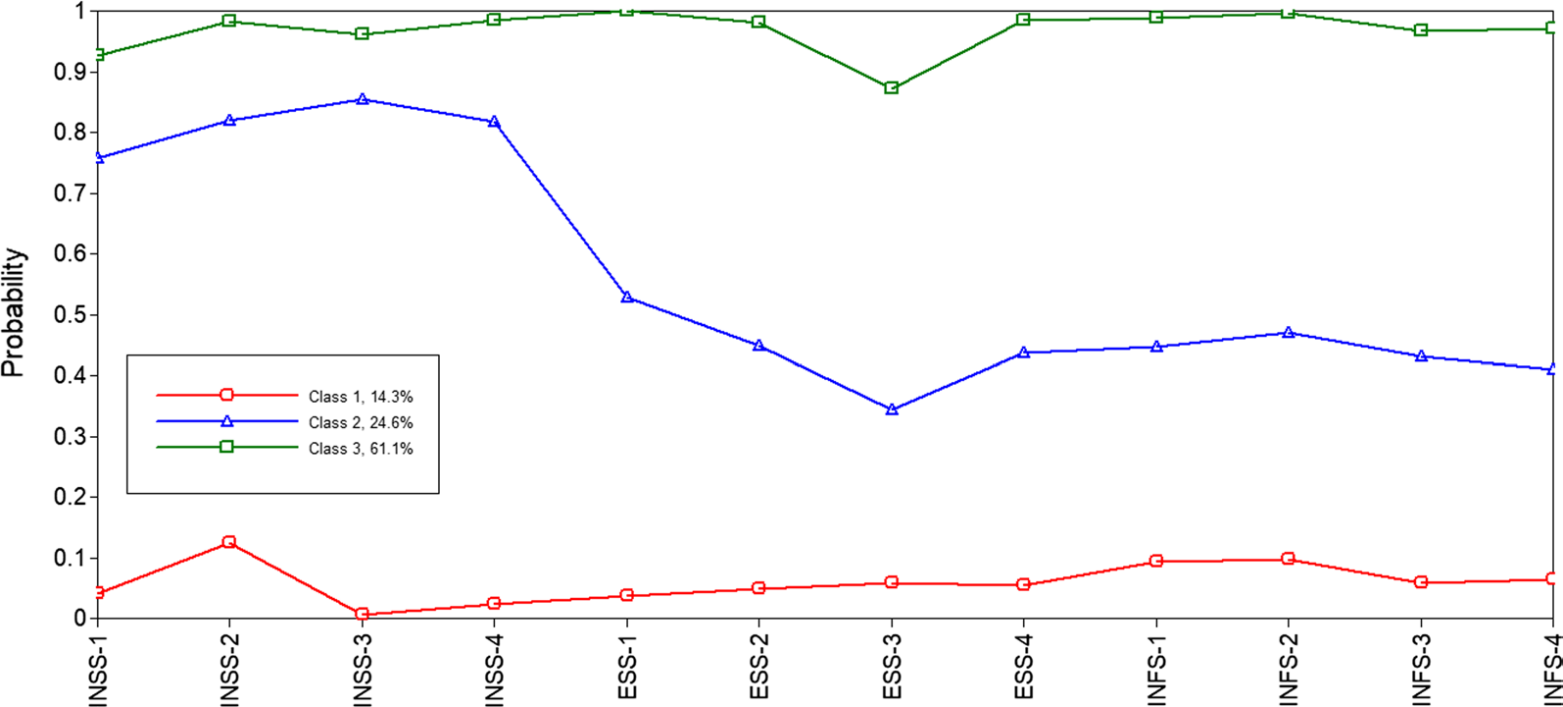
Probability of social relationships classes. INSS 1–4 from the PROMIS-Instrumental Support Short Form, ESS 1–4 from the PROMIS-Emotional Support Short Form, INFS 1–4 from the PROMIS-Informational Support Short Form

The latent class model fit comparison is listed in Table 2. The optimal number of latent classes was determined based on the model fit statistics. The three-class model was found to have relatively low AIC, BIC, and aBIC values; relatively high entropy; and significant LMR and BLRT values. In addition, it provided good class membership classification and the most interpretable information from a clinical perspective. Therefore, the three-class model was selected as optimal for further analysis. The results of item-response probability in the three-class model demonstrated that the average probability of the patients belonging to each potential category ranged from 97.5 to 98.8%, which supported the reliability of the model. Subsequently, we further obtained the response probability graphs of three potential categories on 12 items. Figure 1 depicts the profiles of social relationships. Class 1 accounted for 14.3% (n = 91) of the sample and had a low probability of endorsing all the items. Therefore, we labeled this subgroup the “low social relationships group.” Class 2 accounted for 24.6% (n = 157) of the sample and had a high probability of endorsing the PROMIS-Instrumental Support Short Form items but reported a medium level for the items in the PROMIS-Emotional Support Short Form and the PROMIS-Informational Support Short Form; thus, it was named the “high instrumental support, medium emotional and informational support group.” Patients in Class 3 accounted for approximately 61.6% (n = 390) of the sample and had a high probability of endorsing all the social relationships items and were classified into the “high social relationships group”. The patients in Class 3 comprised most of the participants.

**Table 2:** Latent class model fit comparison

Model	K	Log (L)	AIC	BIC	aBIC	Entropy	LMR *P* value	BLRT *P* value	Probability of classes
1C	26	− 4482.80	9017.61	9133.52	9050.98	–	–	–	1
2C	53	− 2949.01	6004.01	6240.30	6072.03	0.95	< 0.00	< 0.00	0.680/0.320
3C	80	− 2644.35	5448.71	5805.38	5551.39	0.96	0.023	< 0.00	0.143/0.246/0.611
4C	107	− 2545.27	5304.54	5781.58	5441.86	0.97	0.771	< 0.00	0.611/0.035/0.222/0.132
5C	134	− 2472.95	5213.90	5811.31	5385.87	0.96	< 0.00	< 0.00	0.050/0.580/0.117/0.132/0.121
6C	161	− 2432.68	5187.37	5905.16	5394.00	0.96	< 0.112	0.000	0.040/0.131/0.584/0.042/0.099/0.103

*K* number of free parameters, *Log (L)* log likelihood, *AIC* Akaike Information Criterion, *BIC* Bayesian Information Criterion, *aBIC* Adjusted BIC, *LMR* Lo–Mendell–Rubin, BLRT the Bootstrap Likelihood Ratio Test, – not applicable

### Differences in demographic characteristics among the latent classes

As shown in Table 3, a significant difference existed in educational background, employment status, monthly family income, and health insurance characteristics among the latent classes identified by social relationships. Based on the results, logistic regression was performed, considering the potential categories as dependent variables and using the significant demographic variables as independent variables. The Class 1, the “low social relationships group,” was taken as the benchmark for comparison. The odds ratio (OR) was calculated to show the effects of independent variables on the various classes. Characteristics, including an educational background lower than primary school, unemployment, monthly family income of ¥6000 or lower, and a lack of health insurance were set as references. The results in Table 4 indicate that patients with lower education and unemployment were more likely to belong to Class 1. Patients in Class 2 were more likely to be retired (OR = 2.66, *P* < 0.00). In addition, there was a large number of patients with high educational background (OR = 2.35, *P* < 0.00), employment (OR = 3.31, *P* < 0.00), high monthly family income (OR = 2.43, *P* < 0.00), and adequate health insurance (OR = 1.67, *P* = 0.01) in the class with better social relationships (Class 3).

**Table 3:** Differences in demographic characteristics among the latent classes

Variables	Class 1 (n = 91)	Class 2 (n = 157)	Class 3 (n = 390)	*P* value
Age (mean ± SD), years	48.60 ± 9.65	47.21 ± 9.95	48.78 ± 10.52	0.25
Religion				0.15
Yes	9 (9.89)	16 (10.19)	29 (7.44)	
No	82 (90.11)	141 (89.81)	361 (92.56)	
Marital status				0.11
Single	2 (2.20)	3 (1.91)	8 (2.05)	
Married	82 (90.11)	152 (96.81)	365 (93.59)	
Divorced	3 (3.30)	1 (0.64)	11 (2.82)	
Widowed	4 (4.39)	1 (0.64)	6 (1.54)	
Childbearing history				0.53
Yes	86 (94.51)	150 (95.54)	384 (98.46)	
No	5 (5.49)	7 (4.46)	6 (1.54)	
Educational background				< 0.00
Primary school or below	23 (25.27)	46 (29.30)	93 (23.85)	
Secondary school	33 (36.26)	45 (28.66)	41 (10.51)	
High school	25 (27.48)	36 (22.93)	150 (38.46)	
University or above	10 (10.99)	30 (19.11)	106 (27.18)	
Menstrual status				0.08
Premenopausal	51 (56.04)	72 (45.86)	213 (54.62)	
Postmenopausal	40 (43.96)	85 (54.14)	177 (45.38)	
Living style				0.42
Living alone	5 (5.49)	8 (5.10)	5 (1.28)	
Living with family	82 (90.11)	142 (90.45)	377 (96.67)	
Living with others	4 (4.40)	7 (4.45)	8 (2.05)	
Employment status				0.02
Employed	4 (4.40)	23 (14.64)	265 (67.95)	
Retired	15 (16.48)	67 (42.68)	78 (20.00)	
Unemployed	72 (79.12)	67 (42.68)	47 (12.05)	
Monthly family income (¥)				< 0.00
≤ 6000	77 (84.62)	80 (50.96)	163 (41.79)	
> 6000	14 (15.38)	77 (49.04)	227 (58.21)	
Medical insurance				< 0.00
Free medical insurance	2 (2.20)	2 (1.27)	0 (0.00)	
Employee health insurance	32 (35.16)	53 (33.76)	175 (44.87)	
Rural health insurance	52 (57.14)	94 (59.87)	197 (50.51)	
Without health insurance	5 (5.50)	8 (5.10)	18 (4.62)	
Cycle of chemotherapy				0.23
1–3 cycles	42 (46.15)	80 (50.96)	125 (32.05)	
4–6 cycles	22 (24.18)	38 (24.20)	210 (53.85)	
> 6 cycles	27 (29.67)	39 (24.84)	55 (14.10)

**Table 4:** Potential predictors of latent class membership

Characteristics	Class 2 (n = 157)		*P* value	Class 3 (n = 390)		*P* value
	OR	95% C.I		OR	95% C.I	
Educational background						
Primary school or below	Ref			Ref		
Secondary school	0.86	0.56–1.72	0.62	0.77	0.58–2.11	0.56
High school	1.32	0.79–1.91	0.01	1.72	1.34–2.87	< 0.00
University or above	1.67	0.87–2.74	0.64	2.35	1.25–3.36	< 0.00
Employment status						
Unemployed	Ref			Ref		
Retired	2.66	1.62–3.52	< 0.00	1.42	0.51–2.57	0.35
Employed	1.32	0.43–2.67	0.42	3.31	0.68–5.27	< 0.00
Monthly family income						
≤ ¥6000	Ref			Ref		
> ¥6000	1.14	0.33–1.87	0.02	2.43	0.54–3.87	< 0.00
Health insurance						
No	Ref			Ref		
Yes	1.22	0.77–2.13	0.65	1.67	0.63–2.53	0.01

*OR* odds ratio, *95% C.I.* 95% confidence interval of OR

### Differences in anxiety and depression outcomes among the latent classes

The results demonstrated that anxiety and depression were at the reference level (T score: 40–60) but differed between the identified classes. The PROMIS domains are interpreted with a wide range, from a very low to a very high degree. For all domains, the reference level of the T score ranged from 40 to 60. A T score of 30–40 was lower than the reference level, while a T score of 60–70 was higher than the reference level [14]. Our results indicated that the T scores of anxiety (52.17 ± 9.74) and depression (52.83 ± 9.30) in Class 1 were higher than those in Class 2 (51.08 ± 10.55, 52.74 ± 11.24) and Class 3 (48.92 ± 9.80, 48.39 ± 9.58), which were conversely correlated with the scores of social relationships (*P* < 0.05). These results suggested that patients with low social relationships were more likely to experience higher levels of anxiety and depression, which should be given special attention.

## Discussion

To the best of our knowledge, this is the first study to use the LCA approach to identify unobserved subgroups of instrumental, emotional, and informational support in Chinese breast cancer patients undergoing chemotherapy. We identified between-group differences in demographic characteristics, anxiety, and depression outcomes across the identified latent classes.

### Profiles of social relationships

Three classes of social relationships were found: Class 1-low social relationships group; Class 2-high instrumental support, medium emotional and informational support group; and Class 3-high social relationships group. Patients in the high social relationship group constituted the largest proportion of participants in this study (61.1%). The PROMIS-Social Relationships Short Forms scores ranged from low to reference level for patients with breast cancer in this study. However, patients in Class 2 reported a medium level of emotional support and informational support despite having high instrumental support, which supported our original hypothesis, indicating that the patients’ perceptions of instrumental, emotional, and informational support varied and had individual differences in terms of their social relationships level.

### Predictors of latent class membership

This study identified the predictors of patients with potential social relationships profiles, which was particularly relevant in this clinical context. Educational background, monthly family income, health insurance, and employment status showed a significant difference in the identified classes. Patients with high monthly family income and adequate health insurance were more likely to belong to the class with high social relationships. High monthly income has been reported to be associated with a good quality of life in patients with breast cancer [3]. Patients with high income are more likely to have better health insurance and are better able to access information and support regarding their disease and treatment than low-income individuals [3]. Another possible reason for this finding might be selection bias because the patients who voluntarily participated in the study might have sufficient support and be satisfied with their social relationships. Therefore, they were more likely to give relatively positive feedback in the investigation. However, there was a small number of patients with a high educational background and income in the low social relationships group. These patients should be assessed for the exact reason for their low social relationship status, which was expected to be high. Stigma might have an impact on the social relationships of these patients, since they might have high expectations for their social lives [22]. However, cancer and cancer-related treatment prevents them from fulfilling certain social roles and participating in certain activities. Therefore, they should be assessed as to whether they feel stigmatized. In addition, support from significant individuals, such as family members, colleagues, and friends, is an important factor in promoting patients’ social participation [23]. On the other hand, patients with a low educational background and unemployment were likely to have a low level of social relationships. Evidence shows that breast cancer patients with low education or who face unemployment have a high risk of marital conflict and depression, which might impact their social relationships [22, 23]. Unemployed individuals also consequently experienced barriers in maintaining healthy relationship and were highly vulnerable to relational instability and parenting stress, which was associated with low income and stress spillover [24, 25]. In this group, the patients had to consider the impact of the disease and treatment on their family, children, economic situation, and other life domains, which might affect their tendency to participate in social roles and activities [26]. On the other hand, medical insurance has been proven to be associated with patients’ social health status [27]. For patients with high monthly family income and adequate health insurance, their economic burden could be partly reduced.

The results also indicated that high educational background and employment were associated with better social relationships. To some extent, patients with a high educational background seem to have a greater chance of finding employment. The positive effect of employment status on patients with breast cancer has been established in recent studies [28–30]. Olsson et al*.* [31] reported that work adjustments were beneficial for distracting breast cancer patients’ attention from the disease and treatments and contributed to their prognosis. Moreover, patients who engaged in work shortly after breast cancer surgery reported higher life satisfaction than the reference population. In addition, working status and better social relationships were predictors of higher life satisfaction. Vayr et al*.* [32] investigated work adjustment status in patients 1 year after breast cancer diagnosis. It was reported that most of the patients were working, and some returned to work after a period of medical leave. A third of the patients returned to work after completing chemotherapy treatment, while some underwent job adjustment. Work adjustments were found to be protective factors for occupational rehabilitation in this population. Furthermore, working patients could benefit from work adjustments over a longer period of time after diagnosis. Therefore, there existed a link between social relationships and employment in this population. This might be because being employed enhances their social interconnections through day-to-day activities with colleagues and other persons. These results were useful for planning interventions to enhance social relationships. Patients with breast cancer should be encouraged to engage in work within their capacity to improve their ability to participate in social roles and activities.

All the patients in this study were undergoing chemotherapy. Evidence reveals that patients undergoing chemotherapy have multiple uncomfortable symptoms, such as fatigue, nausea, and vomiting, during chemotherapy, and they are more likely to have a vulnerable immune system and damaged body image, which prevents them from maintaining close contact with others [8]. They are more likely to be socially isolated individuals and are less able to deal with the stressors and consequently are at higher risk of adverse health outcomes. Breast cancer and cancer-related treatment are reported to have an impact on the social relationships of patients [4–8]. Compared with the general population, patients with breast cancer are more likely to report lower social relationships [17]. However, significant differences were not found in patients with different cycles of chemotherapy in this study. The average age of the patients was 48.6 years, which might have impacted the results. Young patients have been reported to be vulnerable to cancer treatment, especially in early survivorship [32, 33]. Therefore, future studies are needed to explore the impact of breast cancer and cancer-related treatment in different age groups of Chinese patients. Healthcare providers should identify the factors that prevent patients from maintaining stable social relationships with their family, friends, and other significant persons and encourage them to participate in social roles and activities. Additionally, healthcare providers should also provide support for patients to find high-quality medical resources.

Another significant contribution of this study was the comparison of depression and anxiety in the identified classes. The results demonstrated that the degree of anxiety and depression varied with social relationships patterns. The order of severity for anxiety and depression in the identified classes had an inverse relationship with the level of social relationships, which was in line with previous findings reporting that cancer patients with a low level of support were more likely to suffer from anxiety and depression symptoms [34]. Additionally, women are typically expected to be “a good wife and devoted mother” in Chinese culture [35]. Therefore, Chinese patients might experience high levels of anxiety and depression if they fail to fulfill their roles to maintain family harmony [35]. Additionally, patients in chemotherapy must undergo frequent hospital visits, medical treatment, and follow-up care in addition to experiencing multiple disturbing symptoms, which further result in a drastic reduction in their social behavior. The results provided valuable information to help healthcare providers identify patients with a higher risk of impaired social relationships. Interventional programs are needed for this subgroup and might be useful in identifying individuals with potential social relationships issues and then in assessing depression and anxiety levels, which is also suggested by Shensa et al. [36] and Wang et al. [37].

Understanding the specific profiles and predictors of social relationships in breast cancer patients undergoing chemotherapy could help healthcare providers identify patients at greater risk of social relationships issues. Healthcare providers need to perform routine assessments prior to and following chemotherapy, and an early interventional strategy should be subsequently initiated to address the patients’ unmet social needs. Additionally, when conducting interventions to promote the social relationships of breast cancer patients undergoing chemotherapy, it is important to consider the patient’s educational background, monthly family income, health insurance, employment status, and the level of anxiety and depression. For example, low-income patients should be provided with information and support regarding their disease and treatment as well as ways to improve their health insurance. Unemployed patients should be encouraged to engage in working within their reach. Interventions are needed to reduce patients’ depression and anxiety levels. In addition, family members, colleagues, and friends of patients with breast cancer, who are significant individuals influencing patients’ willingness to participate in social activities, should contribute to supporting the patient and encourage them to be actively involved in social roles and activities during their treatment and rehabilitation.

### Limitation of the study

Several limitations need to be noted in this study. First, the cross-sectional design limited our ability to examine the trajectory of social relationships in patients with breast cancer over time. The results require further testing by longitudinal studies. Second, the data were collected from tertiary hospitals, which might affect the homogeneity of the social relationships profiles. Therefore, the results might not reflect the actual diversity of social relationships for all breast cancer patients undergoing chemotherapy in China.

## Conclusions

Our study identified profiles of instrumental, emotional, and informational support in Chinese breast cancer patients undergoing chemotherapy and identified predictors using LCA. The results indicated that social relationships in this study could be divided into three classes, which were significantly correlated with educational background, monthly family income, health insurance, and employment status. Additionally, patients with low instrumental, emotional, and informational support were more likely to report high levels of anxiety and depression.

## Data Availability

The dataset generated and/or analyzed during the current study is available from the corresponding author on reasonable request.

## Abbreviations

PROMIS: Patient-reported outcomes measurement information system
LCA: Latent class analysis
STROBE: Strengthening the Reporting of Observational studies in Epidemiology studies
Log (L): Log likelihood
AIC: Akaike Information Criterion
BIC: Bayesian Information Criterion
aBIC: Adjusted Bayesian Information Criterion
LMR: Lo–Mendell–Rubin
BLRT: The Bootstrap Likelihood Ratio Test
OR: Odds ratio
